# An optimized secretory expression system and immunogenicity evaluation for glycosylated gp90 of avian reticuloendotheliosis virus

**DOI:** 10.1186/s13567-020-00857-y

**Published:** 2020-10-19

**Authors:** Qing Pan, Jing Wang, Altaf Hussain, Yulong Gao, Hongyu Cui, Kai Li, Xiaole Qi, Changjun Liu, Yanping Zhang, Shide Zhang, Xiaomei Wang

**Affiliations:** 1 Avian Immunosuppressive Diseases Division, State Key Laboratory of Veterinary Biotechnology, Harbin Veterinary Research Institute, The Chinese Academy of Agricultural Sciences, Harbin, 150069 P. R. China; 2 Jiangsu Co-Innovation Center for the Prevention and Control of Important Animal Infectious Disease and Zoonosis, Yangzhou University, Yangzhou, 225009 P. R. China; 3 Departments of Radiology, Second Affiliated Hospital, Harbin Medical University, Harbin, 150001 China

**Keywords:** reticuloendotheliosis virus, glycosylated gp90 protein, vaccine candidate, serum-free culture system, suspension culture

## Abstract

Reticuloendotheliosis is an important immunosuppressive disease, associated with avian reticuloendotheliosis virus (REV) infection, and causes notable economic losses worldwide. Glycoprotein gp90 is an important structural protein of REV, and considered to be the most important immunogenic antigen, which can induce neutralizing antibodies against REV. In this study, an optimized suspension culture system was developed and applied to secretory express the immunogenic surface antigen gp90. To achieve an optimal glycosylation, the gp90 was designed to secretory expressed into the supernatant of the cell culture, which also occurs in the natural protein maturation procedure of REV. Serum-free culture medium was introduced to simplify the purification process and reduce the production costs. Based on the purified glycosylated gp90, an oil-emulsion subunit REV vaccine candidate was developed and evaluated in chickens. The subunit gp90-based vaccine induced fast immune responses, high levels of antibodies (REV-specific antibody, gp90-specific antibody, and neutralizing antibody against REV), and preferential T helper 2 (Th2) (interleukin-4 secretion) not Th1 (interferon-γ secretion) response. Furthermore, the viremia induced by REV infection was significantly reduced in chickens immunized with the glycosylated gp90. Overall, an optimized secretory expression system for glycosylated gp90 was developed, and the glycosylated gp90 obtained in this study retained good immunogenicity and could be an attractive vaccine candidate to protect chickens against REV horizonal infection.

## Introduction

Reticuloendotheliosis virus (REV) is a type-C avian retrovirus [1] that causes tumors, immunosuppression, growth retardation, persistent viremia, or even deaths [2–5] in variable susceptible hosts, including chicken [6], pigeons [7], ducks [8], geese [9], quails [10], and peafowl [11]. The immunosuppression induced by REV infection can reduce the efficiency of vaccines and increase the probability of co-infection with other bacteria or viruses, such as avian influenza virus (AIV) [12], Newcastle disease virus (NDV) [13], fowl adenoviruses (FAdVs) [14], chicken anemia virus (CAV) [15], avian leucosis virus (ALV) [16], Marek’s disease virus (MDV) [17], fowlpox virus (FWPV) [18], or infectious bursa disease virus (IBDV) [19]. Additionally, various vaccines contaminated with REV have been reported. Although notable economic losses worldwide have been induced, there is no efficient commercial vaccine available until now.

As reported, interaction between the env derivative of REV and the cellular component that functions as a receptor for the virus can occur in the endoplasmic reticulum and renders the cell immune to superinfection [20]. Furthermore, in light of reports indicating that the abrogation of interference shown by using the *N*-glycosylation inhibitors [21] and the inability of unglycosylated *E. coli*-synthesized HIV env to bind the CD4 receptor [22] suggested that cotranslational glycosylation is required for the proper conformation to be adopted by these retrovirus proteins for receptor binding. Glycoprotein gp90 [23], a cleavage product of env precursor protein, is an important structural protein of REV which forms the protrusions on the viral surface. gp90 is considered the most important immunogenic antigen, which can induce neutralizing antibodies against REV [24–26]. Several subunit vaccines based on gp90 have been developed and evaluated in previous studies. Non-glycosylated gp90 was expressed in *E. coli*, and the immunogenicity of gp90 coupled with adjuvant CpG-ODN or Poly (I:C) was evaluated [27, 28]. In our previous studies, various DNA vaccines have been developed and provided variable protection to chickens against REV infection, demonstrating that the DNA prime-protein boost vaccination strategy could enhance both humoral and cellular immune responses in chickens [29, 30]. To further improve the immunogenicity, glycosylated gp90 was expressed in *Pichia pastoris*. Glycosylated gp90 showed better immunogenicity than DNA vaccine or prokaryotic non-glycosylated gp90 expressed in *E. coli*, which could fully protect the animals from viremia after REV infection [25], highlighting the importance of the glycosylation for gp90 immunogenicity. Although gp90 expressed from *Pichia pastoris* is glycosylated, the degree of gp90 glycosylation is not enough.

In this study, an optimized serum-free suspension culture system was developed to obtain glycosylated surface antigen gp90 of REV. The recombinant gp90 was expressed and secreted into the supernatant of the serum-free culture, which simplified the purification process. Furthermore, the viremia induced by REV infection was significantly reduced in chickens immunized with glycosylated gp90. Overall, the glycosylated gp90 obtained in this study retained good immunogenicity and could be an attractive vaccine candidate to protect chickens against REV horizonal infection.

## Materials and methods

### Viruses, cells, antibodies, and plasmids

REV strain HLJR0901 [6] (GenBank No: GQ415646) was isolated and stored at the Harbin Veterinary Research Institute (HVRI) of the Chinese Academy of Agricultural Science (CAAS) at -70 °C. Primary chicken embryo fibroblasts (CEFs) were prepared from 10-day-old specific-pathogen-free chicken embryos provided by HVRI [29, 30]. 293F cells were purchased from American Type Culture Collection (ATCC). Gp90-specific monoclonal antibody [31] and p19-2 expression vector [32] were generously provided by Prof. Yunfeng Wang (HVRI, CAAS, China) and Prof. Baoshan Zhang (Vaccine Research Canter, NIH, USA).

### Cell culture

The CEF cells were prepared according to the protocol described before and used for the isolation and propagation of REV. Dulbecco’s modified Eagle’s medium (DMEM, Invitrogen, CA, USA) supplemented with 5% fetal calf serum (FCS), 100 IU/mL penicillin, and 100 μg/mL streptomycin was used for CEF culture. The 293F cells were cultured within 293Pro CD 293 M serum-free culture medium (Peiyuan, Shanghai, China) supplemented with 100 IU/mL penicillin and 100 μg/mL streptomycin in a 125-ml Erlenmeyer flask (Corning, Oneonta, USA) on a shaker (120 rpm) to keep cells floating. Both the cells were incubated at 37 °C / 5% CO_2_.

### Construction of REV gp90 secretory eukaryotic expression plasmid

The full-length gp90 gene was amplified with primers gp90F (5′-CCTCAGGGCGTCGTGGGCTACCCCTTGCAGCAACTTTGG-3′) and gp90R (5′-CTGGAACAGAACTTCCAGCTTATGACGCCCAGCGGT-3′) from the proviral cDNA extracted from CEF cells infected with REV HLJR0901. The PCR product was purified using QIAquick PCR Purification Kit (Qiagen, Hilden, Germany) and cloned into the p19-2 vector by homologous recombination. The constructed plasmid, designated p192-gp90 and containing the open reading frame (ORF) of gp90, signal sequence (SP) and his-tag, was identified by sequencing and purified by the QIAfilter Plasmid Midi Kit (Qiagen, Hilden, Germany). The concentration of plasmid was determined by spectrophotometry (Implen Version 1.3, Munchen, Germany) at 260 nm.

### Optimization of the secretory expression system for REV-gp90

When the concentration of 293F cells reached 2 × 10^6^ cells/mL, difference concentrations of p192-gp90-His was transfected with X-tremeGENE HP DNA Transfection Reagent (Roche, Mannheim, Germany) into the suspended 293F cells. The cell supernatant was harvested at each 24 h post transfection by centrifugation until 120 h. The gp90 in the supernatant was harvested by centrifuged at 10,000 × *g* for 30 min to remove cell debris and subsequently purified by Ni Sepharose Excel resin (GE Healthcare, Pittsburgh, USA), following the manufacturer’s recommended protocol. The expression of recombinant proteins was detected by SDS-PAGE and western blot analysis, using mouse anti-gp90-specific monoclonal antibody with a concentration of 1:1000 for 1 h at room temperature. Furthermore, 2 μL (1 mU) glycopeptidase F (Takara Bio, Otsu, Japan) was incubated with 25 μg purified gp90 protein for 20 h at 37 °C according to the manufacturer’s introduction. The cleavage product of the purified gp90 protein was identified by SDS-PAGE.

### Immunization, challenge, and samples collection

The purified gp-90 was emulsified with white oil (Weike, Harbin, China) as a sustained releasing adjuvant to a final concentration of 80 μg/mL. Thirty 3-week-old SPF chickens were randomly divided into three groups, including 10 birds in each group. Chickens were immunized twice at 2-week intervals intramuscularly with 0.5 mL vaccine containing 40 μg purified gp90 per chicken. Birds in the negative control group and challenge control group were administered with 0.5 mL phosphate buffer saline (PBS) at the same time points. Two weeks after the second immunization (IM), animals were challenged with 10^4^ TCID_50_ of HLJR0901 in 0.5 mL PBS intraperitoneally. Serum samples were collected weekly after vaccination for antibody and cytokine assays. Anticoagulated blood was collected 7 days post infection (dpi) for viremia detection and virus quantification.

### REV-specific antibody and cytokine release assay

Serum samples were collected weekly for antibody and cytokine assay. The REV-specific antibodies in the serum were detected using a commercial enzyme-linked immunosorbent assay (ELISA) kit (IDEXX, Westbrook, USA) according to the manufacturer’s instructions. The relative level of antibody titer in the unknown was determined by calculating the sample to positive (S/P) ratio as [(mean of sample optical density)-(mean of negative control optical density)]/[( mean of positive control optical density)-( mean of negative control optical density)]. End-point titers were calculated with the equation: log10 titer = 1.09 (log10 S/P) + 3.36 [25]. The cytokines (IL-4 and IFN-γ) were also analyzed using commercial cytokine ELISA kits according to the manufacturer’s instructions (Cloud-Clone, Houston, TX, USA).

### Detection of gp90 antigen-specific antibody assay

Microtiter plates (Costar, Kennebunk, USA) were coated with purified recombinant gp90 protein (2 μg/mL, 100 μL/well) at 4 °C overnight and incubated with test sera diluted within 1:500 at 37 °C for 1 h. For the secondary antibody, an HRP-conjugated goat anti chicken IgG antibody (Southern Biotech., Birmingham, USA) was used at 37 °C for 1 h. Following incubation with tetramethylbenzidine substrate (TMB, Tiangen, Beijing, China) at room temperature (RT) for 15 min, the reaction was stopped with 0.5 M sulphuric acid and the optical density of wells in the plates was measured at a wavelength of 450 nm (OD_450nm_).

### Neutralizing antibody assay

The LMH cells were plated in 96-well plates (Costar, Kennebunk, USA) with a density of 1 × 10^6^ cells/mL and 100 μL/well. Then, the collected serum, inactivated at 56 °C for 30 min, was submitted to neutralize 100 TCID_50_/100 μL of strain HLJR0901 within constant serum dilutions. The plates were incubated at 37 °C in 5% CO_2_ for another 7 days and investigated by indirect immunofluorescence assay (IFA).

### Protection efficacy against challenge with REV

At 7 dpi with REV, the anticoagulated blood was collected in heparinized tubes. Then the samples were submitted for viremia detection and virus quantification according to the methods described previously [33] with several optimizations. Genomic DNA was extracted using the Kit (Corning, USA) and detected by a real-time PCR. Briefly, Real-time PCR was performed with a LightCycler 480 real-time thermocycler (Roche, Rotkreuz, Switzerland). The primers were designed at *gp90* as follows: forward primer 5′-AAGAATCTGTGCGTGAAAG-3′, and reverse primer 5′-TAAGGACCTGGTGAGTAGC-3. The primers were designed at *ovo* as follows: orward primer 5′-CACTGCCACTGGGCTCTGT-3′, and reverse primer 5′-GCAATGGCAATAAACCTCCAA-3′. The samples were analyzed by qPCR performed using SYBR Green Relative PCR master mix (Toyobo, Osaka, Japan). Quantitative PCR was performed under the following conditions: 95 °C for 30 s for initial denaturation, followed by 40 cycles for 5 s at 95 °C, 10 s at 60 °C, 15 s at 72 °C and collection of the PCR product. All controls and infected samples were examined in triplicate on the same plate. The relative REV loading was calculated according to the method of 2^−△△Ct^. Furthermore, the plasma samples were inoculated on CEF cells following incubation for another 7 days, and the cultures were checked for the evidence of virus by IFA with anti-gp90 monoclonal antibody.

### Statistical analysis

Statistical analyses were performed using the GraphPad Prism package (GraphPad Software, La Jolla, CA, USA). The statistical significance of the difference between two groups was evaluated by Student’s *t*-test and between more than two groups by one-way ANOVA. Differences were considered to be significant at *p < *0.05* or **p < *0.01*.

## Results

### Development and application of the optimized secretory expression system for recombinant REV-gp90

**Figure 1:**
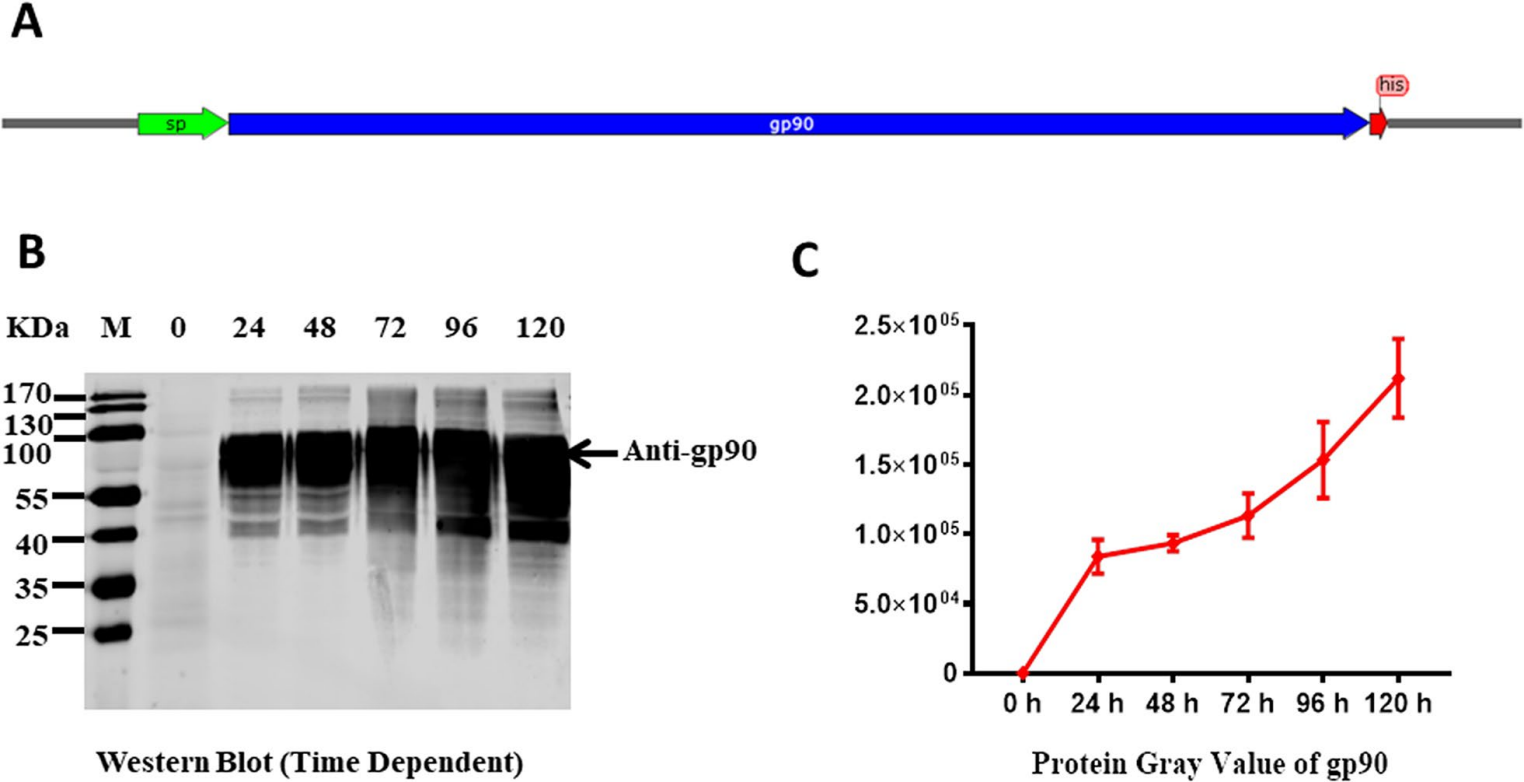
**Construction and expression of recombinant gp90 of REV. A** The ORF of recombinant gp90 contained the whole length gp90 (1083 bp), the signaling peptide (87 bp) at the N-terminus, and a his-tag at the C-terminus. **B** Western blot identification of recombinant gp90 with anti-gp90 mAb at different expression time points. **C** The gray value assay for expressed gp90 at variable time points.

**Figure 2:**
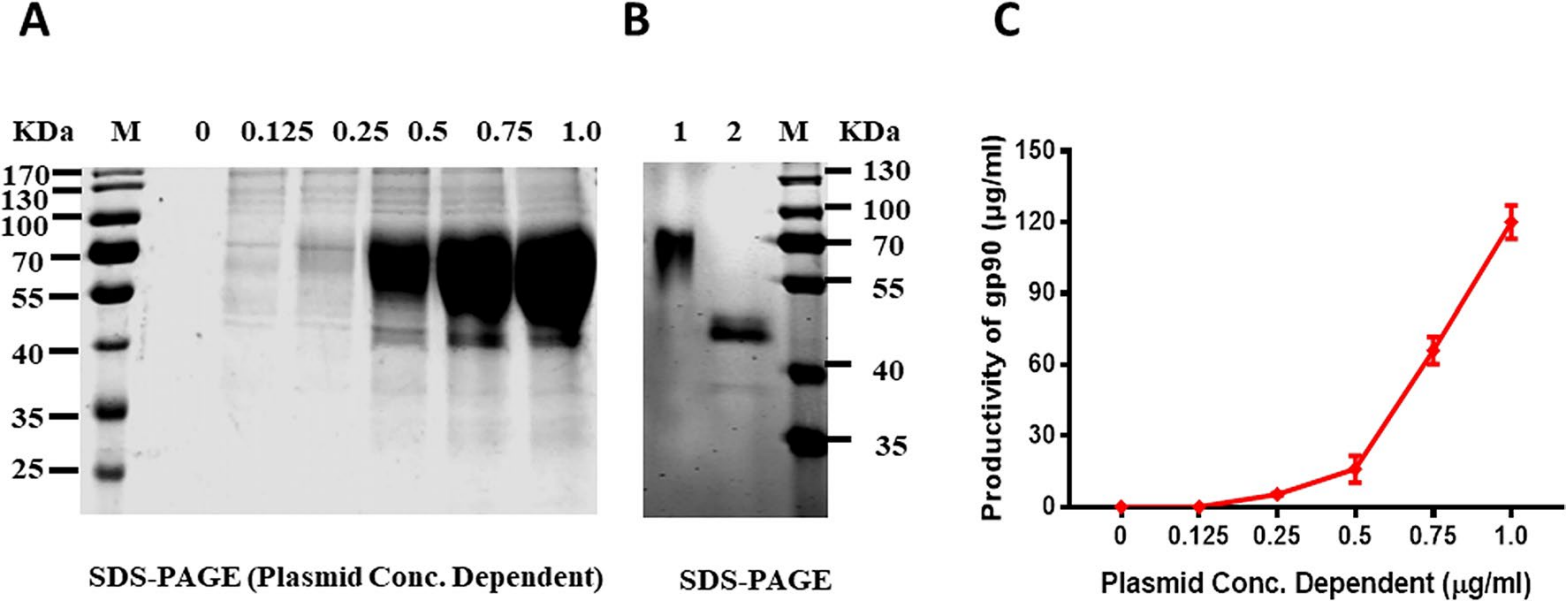
**Concentration-dependent manner between the recombinant gp90 productivity and transfected p192-gp90. A** SDS-PAGE for the purified gp90 from serum-free culture supernatant. The purified gp90 showed high purity and glycosylation. **B** Glycosylation analysis of purified recombinant gp90 protein. Lane 1, purified gp90 protein from serum-free culture supernatant; lane 2, glycopeptidase F treated recombinant gp90 protein; lane 3, prestained protein standards. **C** The expression efficacy of gp90 showed a concentration-dependent manner with the transfected p192-gp90, and the highest concentration of gp90 was 120 μg/mL.

The gp90 gene of REV was successfully amplified from the cells infected with HLJR0901 strain, and then cloned into the p19-2 vector for constructing the recombinant gp90 protein. The recombinant gp90 contained the whole ORF of gp90 (1083 bp), the signaling peptide (87 bp) at the N-terminus, and a his-tag at the C-terminus (Figure 1A). The cell supernatant was harvested at 0, 24, 48, 72, 96 and 120 h post transfection, and submitted to western blot assay using mAb against the gp90 protein (Figure 1B). The results showed a highest productivity appeared at 120 h by a gray value assay (Figure 1C). Furthermore, different concentrations of plasmid (p192-gp90) was transfected into 293F cells to select an optimal factor. The separately expressed products were harvested at 120 h post transfection and purified using Ni Sepharose Excel resin, and subsequently submitted to SDS-PAGE. The purified gp90 from serum-free culture supernatant showed high purity and glycosylation (Figure 2A). The expression efficacy of gp90 showed a concentration-dependent manner with the transfected p192-gp90, and the highest concentration of gp90 was 120 mg/l (Figure 2C). Furthermore, a glycosylation assay was conducted and the gp90 protein became significantly smaller (Figure 2B) after incubated with a glycopeptidase F, which specifically cleaving the N-linked glycosylation.

### REV-specific and gp90-specific antibody responses of vaccinated chickens

**Figure 3:**
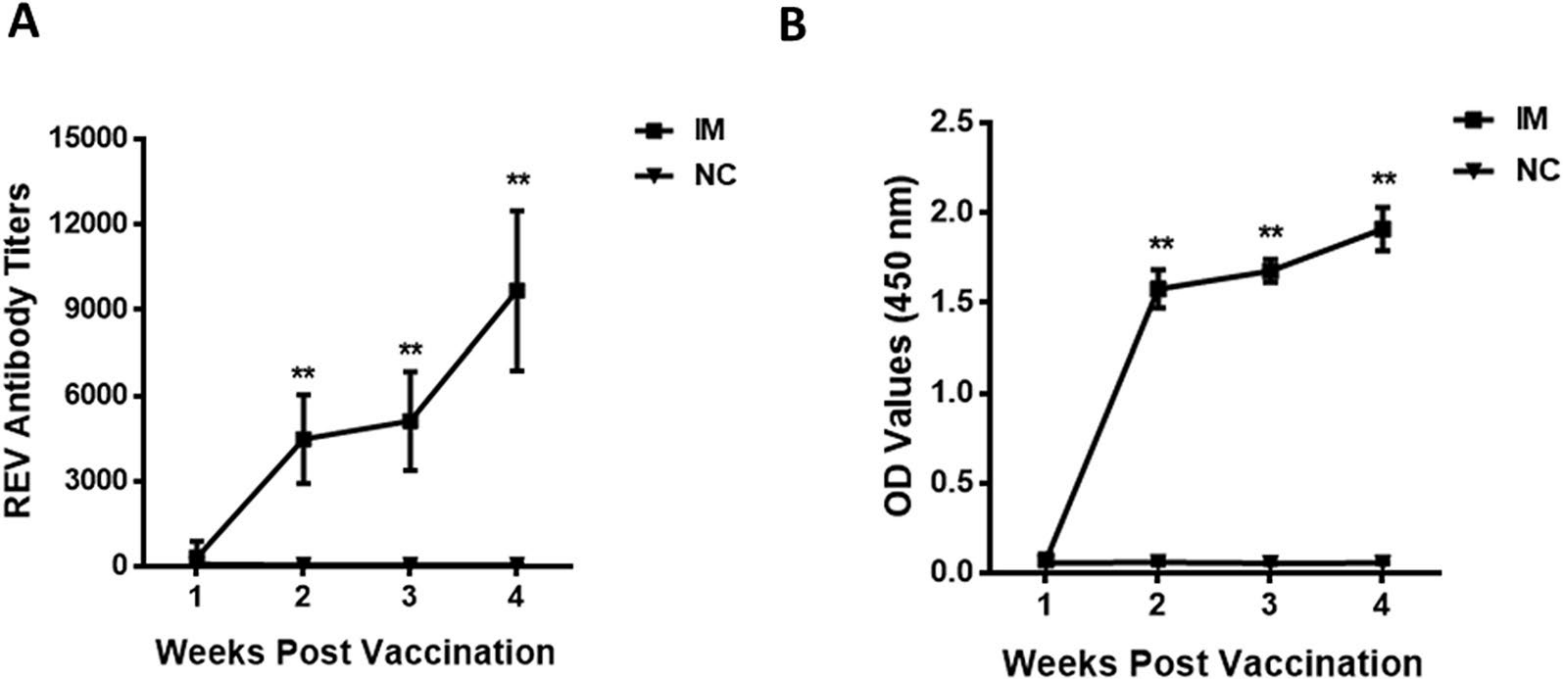
**REV-specific antibody titers-specific antibody responses detected by indirect ELISA induced from 1 to 4 weeks post the first inoculation, and the booster inoculations were conducted at the second week post first inoculation. A** REV-specific antibody titers detected by a commercial REV antibody test kit (IDEXX, Westbrook, USA), serum with titer of higher than 1076 was considered positive for REV antibody. **B** Gp90-specific antibody responses detected by the gp90 protein-based indirect ELISA, OD_450nm_ values higher than 0.19 were considered positive for gp90 antibody. Serum samples were collected weekly, and REV-specific and gp90-specific antibodies were detected. The recombinant gp90 protein induced detectable REV-specific and gp90-specific antibodies in chickens at the second week post first inoculation, which was significantly higher (**p < *0.01*) than negative control group. And the magnitude of antibody response was time dependent.

REV-specific antibody responses elicited after IMs were measured by titrating the serum of the control and vaccinated chickens against REV by ELISA. Chickens in the negative control group were negative for REV antibodies throughout the experiment. The recombinant gp90 protein induced detectable antibodies in chickens at two weeks post first inoculation (Figure 3A), and the mean REV-specific antibody titer in the sera of chickens immunized with the recombinant gp90 protein was 4470, which was significantly higher than that in the negative control group (77, **p < *0.01*). The magnitude of antibody response was time dependent, and the antibody titers were significantly higher (**p < *0.01*) at 14 dpi than 7 dpi both for single and double IM. The double IM group had significantly higher antibody responses than the single IM group at 7 dpi (**p < *0.01*) and 14 dpi (**p < *0.01*). The gp90 antigen-specific antibody was detected by the gp90 protein-based indirect ELISA simultaneously, showing that the vaccine could also cause high levels of gp90-specific antibody at two weeks post first inoculation (Figure 3B), and the tendency was consistent with the REV-specific antibody test results.

### Neutralizing antibody against REV

**Figure 4:**
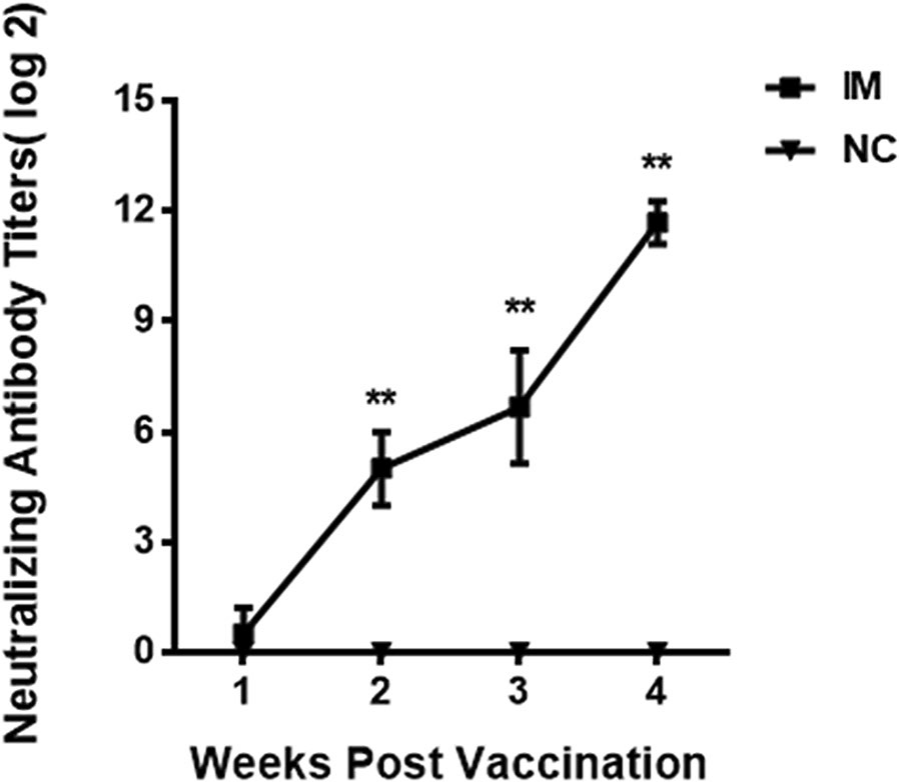
**Neutralizing antibody titers detected by IFA.** The recombinant gp90 protein induced detectable neutralizing antibodies in chickens at the second week post first inoculation. The neutralizing antibody titers are presented as mean ± standard deviation. Significant differences between experimental group and negative control group were evaluated at (**p < *0.01*) of immunized animals. And the magnitude of neutralizing antibody response was time dependent.

The serum from the negative control group and the vaccinated group neutralized with 100 TCID_50_ of strain HLJR0901 was detected using IFA mediated by the MAb of REV at 7 dpi. The results showed that the recombinant gp90 protein induced detectable neutralizing antibodies in chickens at the second week post first inoculation, and the mean neutralizing antibody titer in the sera of chickens was 2^5^, whereas that in the negative control group was negative. The neutralizing antibodies of chickens in the single IM (**p < *0.01*) and double IM (**p < *0.01*) groups were significantly higher than those in the negative control group at 7 dpi and 14 dpi (Figure 4).

### Cytokine production of SPF chickens

**Figure 5:**
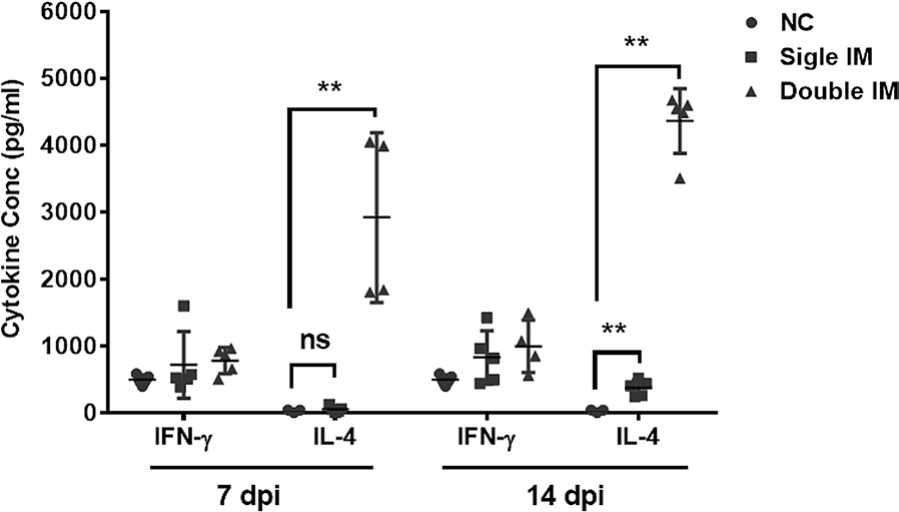
**REV specific T helper 1 (Th1)/Th2 cytokine responses induced 7 days and 14 days post single and double immunization (IM).** Th1 (interferon (IFN)-γ) and Th2 (interleukin (IL)-4) cytokines in serum of chickens were detected by ELISA. A strong Th2 response was induced in the vaccinated chickens. Significant differences between Th1 and Th2 cytokines (IFN-γ and IL-4) were evaluated at (*p < *0.05*) or (**p < *0.01*).

The cellular immune responses were evaluated by analyzing cytokine production (Figure 5). IL-4 (Th2 response) and IFN-γ (Th1 response) in the serum of chickens were detected 7 and 14 days after the single and double IM by ELISA, which showed that the IL-4 concentrations of chickens in the single IM (**p < *0.01*) and double IM (**p < *0.01*) groups were significantly higher than those in the negative control group at 14 dpi. The IL-4 concentrations of chickens in the double IM group were significantly higher (**p < *0.01*) than those in the negative control group at 7 dpi, but there was no difference (*p* > *0.05*) between the IL-4 concentrations in the negative control group and single IM group at 7 dpi. The IL-4 levels in the double IM groups were significantly higher (*0.01* < *p < *0.05*) than those in the single IM group at 14 dpi, whereas those in the double IM groups were significantly higher (**p < *0.01*) than those in the single IM group at 7 dpi. For the IFN-γ assay, there was no difference (*p* > *0.05*) between the two immunized groups and the control group at any detected time points.

### Protective efficacy against viremia induced by REV

**Figure 6:**
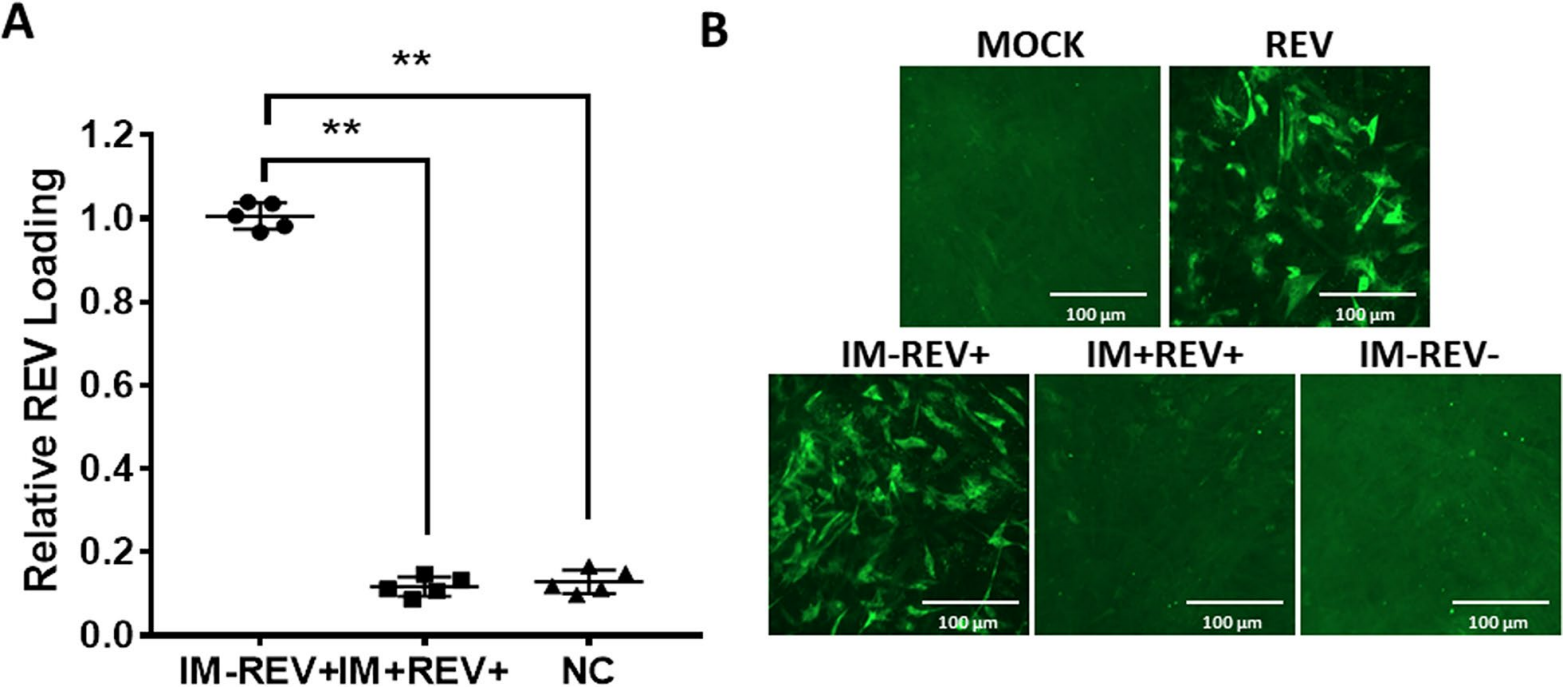
**Protection efficacy according to the viremia against challenge with REV and IFA of viremia in CEF cells for the chickens in different groups at the second week after being challenged. A** Chickens were challenged with 10^4^ TCID_50_ of REV HLJR0901 at 4 weeks post immunization and viremia was detected at 7 days post challenge. The viral load of REV challenge group was significantly higher (**p < *0.01*) than the recombinant gp90 protein vaccinated and the infection control group. **B** Mock: negative control; REV: positive control (cell supernatant containing REV); IM-REV+ : plasma samples from the challenged chickens in the control group; IM+ REV+ : plasma samples from the challenged chickens in the recombinant gp90 group; IM-REV−: plasma samples from SPF chickens.

To evaluate the protective efficacy according to the viremia of the inactivated vaccine, the chickens were infected with 10^4^ TCID_50_ of REV HLJR0901 in 0.5 ml PBS via intraperitoneal administration and observed for 7 days. The results showed that the vaccine induced protection (Figure 6A) against viremia caused by REV infection. Firstly, REV viremia was not detected in any of the samples taken from the negative control animals. At 7 dpi with REV, viremia was observed in tested chickens challenged with REV, with no evident difference in viral load between the recombinant gp90 protein vaccinated and negative control birds. The viral load of the group challenged with REV was significantly higher (**p < *0.01*) than the recombinant gp90 protein vaccinated and the infection control groups. The protective efficacy of the vaccines was confirmed by IFA with CEF cells that were treated with the plasma samples from the REV-infected chickens. Intensive fluorescence was observed in the REV challenge group, whereas no fluorescence was found in the recombinant gp90 protein vaccinated and negative control groups (Figure 6B), which corresponded with the real-time PCR assay results.

## Discussion

REV causes immunosuppression in infected chickens, resulting in poor immune responses to other vaccines and increased susceptibility to concurrent or secondary bacterial or viral infections, leading to serious economic losses to the poultry industry [34, 35]. However, there is currently no efficient commercial vaccine available. The gp90 protein of REV is a major component of the viral envelope and is responsible for eliciting virus-neutralizing and protective antibodies. As reported, gp90 is a major candidate antigen for vaccines and serological diagnosis [24, 26]. As reported, several studies have been conducted on REV vaccines based on gp90. DNA vaccines and prokaryotic expressed gp90 subunit vaccine in *E. coli* have been developed and provided partial protection [25, 27, 28]. To further improve the immunogenicity, glycosylated gp90 expressed in *Pichia pastoris* showed better immunogenicity than non-glycosylated gp90 [25, 29] in our previous studies.

However, the degree of gp90 glycosylation expressed from *Pichia pastoris* is not enough. Thus, an optimized serum-free suspension culture system for the secretory glycosylated gp90 of REV was developed in this study. The gp90 was highly glycosylated within the secreting process, maintained essential modifications of eukaryotic proteins, which is present in the natural state, and retained good immunogenicity. Glycosylation assay showed that the gp90 protein expressed in our study was heavily glycosylated and the type of glycosylation was mainly N-linked glycosylation. Meanwhile, the recombinant gp90 was expressed and secreted into the suspension of the serum-free cell culture supernatant, significantly simplifying the purification process and reducing the cost of the vaccine candidate. Furthermore, this secretory system was able to get an ideal yield of 120 mg of purified gp90 in one liter of cell supernatant of serum-free culture. The system developed in this study provided a powerful tool to express proteins with modifications (not only glycosylation) for the functional and structural studies.

As reported by Yuan et al. [28], the mean titers of the REV-antibody were positive at the third week post first inoculation, and the antibody positive ratio was 23% (3/13) at the second week post first inoculation in the gp90 group. However, the mean antibody titers of the REV-antibody reached 4470, and 100% (8/8) positive results were obtained at the second week post first inoculation following gp90 immunization. Our results show that the recombinant gp90 protein further improved the titers of the REV-specific antibody and the antibody positive ratio, which is conducive for use as a vaccine. Moreover, gp90 antigen-specific antibodies were detected by the gp90 protein-based indirect ELISA, which showed that the vaccine could induce high levels of gp90-specific antibody against REV at the second week post first inoculation. The results of the neutralization test in vitro confirmed that the vaccine could induce high levels of neutralizing antibody against REV, and the neutralization depends on the titers of REV-specific and gp90-specific antibody.

The recombinant gp90 protein vaccine developed in this study can induce high levels of IL-4 not IFN-γ, suggesting that a strong Th2 differentiation and in cell expansion occurred. Consistent with previous reports, protein vaccination can induce an immune response that has a Th2 type bias [36–38]. High levels of IL-4 secretion further stimulate B cells to differentiate into plasma cells, thus participating in protective humoral immune response. Furthermore, the viremia of REV infection was also monitored after the challenge, and our results showed that viremia induced by REV infection was significantly reduced in chickens immunized with glycosylated gp90 and full protection against viremia was achieved. Taken together, the present findings warrant further investigation in the research and development our proposed vaccine against REV infection.

In this study, an optimized serum-free suspension culture system was developed to obtain glycosylated surface antigen gp90 of REV. The recombinant gp90 was expressed and secreted into the supernatant of the serum-free culture with high yield and heavy glycosylation. The type of glycosylation was further identified as mainly N-linked glycosylation. The glycosylated gp90 was submitted to formulat an oil-emulsion vaccine, which induced fast Th2 immune responses and high levels of antibodies against REV infection. Moreover, the vaccine significantly reduced the viremia induced by REV infection. For the first time, a novel secretory glycosylated gp90 subunit vaccine has been developed and is proposed as an attractive candidate to protect chickens against REV horizonal transmission and reduce the economic losses to the poultry industry.

## Data Availability

The datasets used and/or analysed during the current study are available from the corresponding author on reasonable request.

## Abbreviations

AIV: avian influenza virus
ALV: avian leucosis virus
CAV: chicken anemia virus
dpi: days post infection
FAdVs: fowl adenoviruses
FWPV: fowlpox virus
IBDV: infectious bursa disease virus
MDV: Marek’s disease virus
NDV: Newcastle disease virus
ORF: open reading frame
REV: reticuloendotheliosis virus
SP: signal sequence
